# Transpapillary Placement of a Multihole Covered Metal Stent Using a 5.9‐Fr Delivery System Through a Forward‐viewing Endoscope in Malignant Biliary Obstruction With Type I Duodenal Stenosis

**DOI:** 10.1002/deo2.70392

**Published:** 2026-08-02

**Authors:** Kengo Matsumoto, Daisuke Kondo, Satoru Okabe, Naoto Osugi, Akira Doi, Masashi Yamamoto, Shiro Hayashi, Koji Fukui, Tsutomu Nishida

**Affiliations:** ^1^ Department of Gastroenterology Toyonaka Municipal Hospital Toyonaka Japan

**Keywords:** duodenal obstruction, forward‐viewing endoscope, malignant biliary obstruction, pancreatic cancer, self‐expanding metal stents

## Abstract

Concurrent malignant biliary obstruction (MBO) and type I duodenal stenosis represent a technically demanding scenario, as luminal narrowing frequently precludes passage of a standard side‐viewing duodenoscope and limits conventional transpapillary drainage. Although endoscopic ultrasound‐guided biliary drainage and percutaneous transhepatic biliary drainage are established alternative approaches, transpapillary drainage remains desirable when papillary access is achievable. We report a 66‐year‐old woman with stage IV pancreatic cancer who presented with obstructive jaundice and tumor‐related type I duodenal stenosis (i.e., obstruction proximal to the papilla). A side‐viewing duodenoscope failed to traverse the stenosis; however, a standard forward‐viewing endoscope (GIF‐Q260J) successfully reached the major papilla. Selective biliary cannulation was achieved, and a covered multihole self‐expandable metal stent (SEMS) (HANAROSTENT Biliary Multihole Benefit) was deployed across the distal biliary stricture using an ultra‐slim 5.9‐Fr delivery system compatible with the 3.2‐mm working channel. Stent placement was technically successful without adverse events, resulting in prompt resolution of cholangitis and enabling the initiation of second‐line chemotherapy. This case highlights a practical and reproducible strategy that expands the therapeutic capability of standard forward‐viewing endoscopes. The combination of a slim delivery system and covered multihole SEMS may provide a physiological alternative to non‐transpapillary drainage in selected patients with MBO and advanced type I duodenal stenosis.

## 1 Introduction

Concurrent malignant biliary obstruction (MBO) and duodenal stenosis are common in advanced pancreatobiliary cancers [1, 2]. When duodenal stenosis occurs proximal to the major duodenal papilla (type I), advancing a standard side‐viewing duodenoscope is often technically unfeasible, making conventional transpapillary biliary drainage challenging [2, 3].

Although endoscopic ultrasound‐guided biliary drainage (EUS‐BD) and percutaneous transhepatic biliary drainage (PTBD) are established alternatives, they require specific expertise and carry risks such as bile leakage and related complications [2, 4].

A slim forward‐viewing endoscope (FVE) can sometimes traverse type I stenosis to reach the papilla [2, 5]. However, its application for biliary stenting has been limited by the absence of an elevator and a small working channel (typically 2.8–3.2 mm), which cannot accommodate conventional self‐expandable metal stent (SEMS) delivery systems (usually 8.5 Fr). Consequently, most previous reports of FVE‐mediated drainage have been limited to plastic stents or nasobiliary tubes [2, 5]. Although some studies have reported SEMS placement using 6‐Fr delivery systems through an FVE [6], these have mainly involved uncovered stents. The use of covered SEMS, which is important for preventing tumor ingrowth, remains challenging through narrow channels because of the larger delivery profiles of conventional systems.

Recently, a novel covered multihole SEMS with an ultra‐slim 5.9‐Fr delivery system has become available. Herein, we report successful transpapillary placement of a covered multihole SEMS using this 5.9‐Fr delivery system through a standard FVE in a patient with MBO complicated by type I duodenal stenosis, and discuss the clinical utility of this approach in the context of the current literature.

## 2 Case Report

A 66‐year‐old woman was found to have a 35‐mm mass in the head of the pancreas in February 2025. EUS‐guided fine‐needle aspiration (EUS‐FNA) confirmed the diagnosis of adenocarcinoma. Gadolinium ethoxybenzyl diethylenetriamine pentaacetic acid–enhanced magnetic resonance imaging revealed small liver metastases, and she was diagnosed with stage IV pancreatic cancer. The patient received eight courses of gemcitabine plus nab‐paclitaxel.

In October 2025, she was admitted for obstructive jaundice due to tumor progression. Contrast‐enhanced computed tomography revealed duodenal stenosis caused by the tumor (Figure 1), and the passage of a side‐viewing duodenoscope (JF‐260 V; Olympus Medical Systems, Tokyo, Japan) across the stenosis was barely achievable. Endoscopic retrograde cholangiopancreatography (ERCP) with a small endoscopic sphincterotomy was performed, and a SEMS (HANAROSTENT Biliary Multihole Benefit, 8 mm × 60 mm) with a 5.9‐Fr delivery system was placed to relieve the distal MBO. The following day, she developed post‐ERCP pancreatitis (PEP). The SEMS was immediately removed and replaced with two 7‐Fr, 7‐cm double‐pigtail biliary plastic stents (Olympus Medical Systems), after which pancreatitis promptly improved.

**FIGURE 1:**
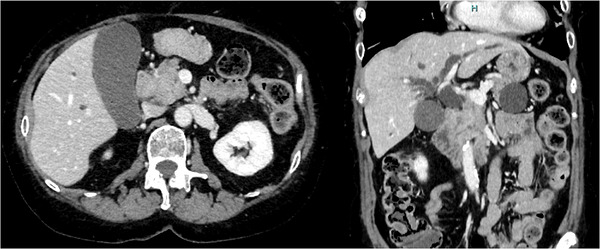
Contrast‐enhanced computed tomography (CE‐CT) at admission. CE‐CT shows a pancreatic head mass causing distal biliary obstruction with upstream bile duct dilatation and concomitant duodenal stenosis due to tumor invasion.

On day 7, inflammation worsened due to the rupture of a pancreatic cyst, which was associated with increased intrapancreatic ductal pressure. Repeat ERCP was performed, and a 5‐Fr, 8‐cm single‐pigtail pancreatic duct stent (Zimmon; Cook Medical, Bloomington, IN, USA) was placed, resulting in clinical improvement.

On day 16, the cholangitis recurred. Repeat ERCP using a side‐viewing duodenoscope was considered; however, the progression of tumor‐related duodenal stenosis precluded the passage of the JF‐260 V. In contrast, a FVE (GIF‐Q260J; Olympus Medical Systems) was used to traverse the stenosis. Biliary cannulation was achieved under direct visualization using a Zemex articulating cannula (Zeon Medical Inc., Tokyo, Japan). After the removal of the biliary plastic stents, a 6‐Fr ENBD tube (Flexima; Boston Scientific, Marlborough, MA, USA) was inserted. The patient remained able to tolerate oral intake; therefore, duodenal intervention was not considered necessary at that time.

After improvement of cholangitis, permanent drainage was planned on day 20. Because the passage of the side‐viewing duodenoscope remained difficult, the procedure was performed again using the FVE. Following removal of the ENBD tube, biliary cannulation was achieved within 1 min using a Zemex articulating cannula, and a 0.025‐inch guidewire (VisiGlide2; Olympus Medical Systems) was advanced. Although the GIF‐Q260J has a 3.2‐mm working channel, the 5.9‐Fr delivery system passed smoothly through it without substantial resistance. A 6 mm × 60 mm covered multihole SEMS was successfully deployed across the distal biliary stricture under endoscopic (Figure 2) and fluoroscopic (Figure 3) guidance.

**FIGURE 2:**
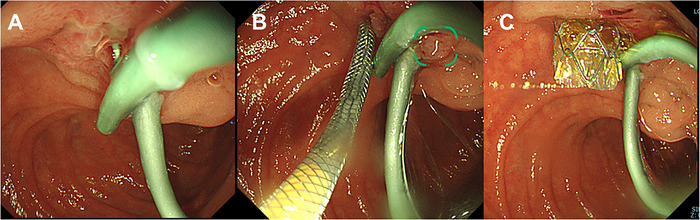
Endoscopic images of the transpapillary biliary stenting procedure. (A) Endoscopic view of the major duodenal papilla obtained using a standard forward‐viewing endoscope after traversing the type I duodenal stenosis. Biliary cannulation is performed with an articulating cannula. (B) Insertion of the ultra‐slim 5.9‐Fr delivery system into the bile duct under direct endoscopic visualization. (C) Final endoscopic view showing the successfully placed covered multihole self‐expandable metal stent (SEMS) at the papilla.

**FIGURE 3:**
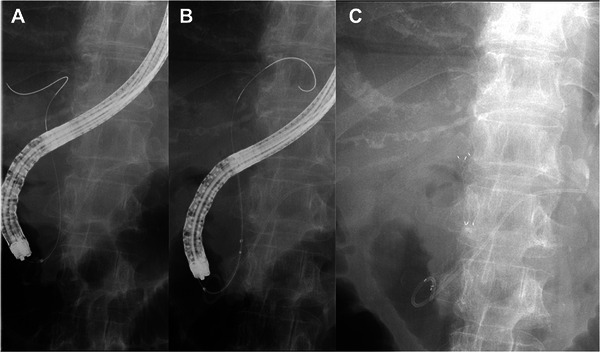
Fluoroscopic images showing transpapillary biliary stenting via a standard forward‐viewing endoscope. (A) Biliary cannulation achieved using an articulating cannula and a 0.025‐inch guidewire through a standard forward‐viewing endoscope (GIF‐Q260J). (B) Advancement of the ultra‐slim 5.9‐Fr delivery system through the 3.2‐mm working channel of the endoscope. (C) Successful deployment of a 6 mm × 60 mm covered multihole self‐expandable metal stent (SEMS) across the distal biliary stricture.

After SEMS placement, neither PEP nor cholangitis recurred. Second‐line chemotherapy with liposomal irinotecan plus 5‐fluorouracil was initiated on day 25, and the patient was discharged in a stable condition. No recurrent biliary obstruction, stent dysfunction, or cholangitis was observed during 5 months of follow‐up.

## 3 Discussion

This case demonstrates the feasibility of physiological transpapillary biliary drainage with SEMS placement by combining a standard FVE with an ultra‐slim delivery system in a patient with MBO complicated by type I duodenal stenosis, in whom the passage of a side‐viewing duodenoscope was not possible.

A FVE is advantageous for type I duodenal stenosis. Advancing a side‐viewing duodenoscope (e.g., JF‐260 V) is often difficult when the stenosis is proximal to the papilla, partly because of its relatively large outer diameter and rigid‐tip structure. In contrast, a conventional FVE (e.g., GIF‐Q260J) has a smaller distal‐end diameter and is more flexible, allowing easier access to the papilla. In addition, direct visualization during advancement may help reduce the risk of perforation compared with blind advancement. Although tumor‐related anatomical distortion and prior EST may have facilitated biliary cannulation in this case, previous reports have also demonstrated successful forward‐viewing cannulation in similar settings. The use of an articulating cannula may also have facilitated biliary cannulation by improving alignment with the biliary axis in the absence of an elevator.

In addition, this approach may offer practical advantages over other drainage techniques. When transpapillary access with a side‐viewing duodenoscope is not feasible, EUS‐BD or PTBD is generally considered as an alternative approach [3, 7]. Although EUS‐BD has become increasingly standardized and widely adopted, it still requires dedicated expertise and equipment [2, 8]. EUS‐BD and PTBD are valuable alternative drainage techniques; however, physiological transpapillary drainage remains desirable when papillary access can be achieved. We do not propose this approach as a universal first‐line strategy; however, it may be considered in selected patients in whom a FVE can traverse the stenosis and transpapillary access is achievable or has already been established.

Another important technical consideration is that the working‐channel diameter of the standard FVE represents a major barrier to SEMS placement. Many widely available FVEs have working channels of approximately 2.8–3.2 mm. Conventional SEMS delivery systems cannot be advanced through this channel or can be advanced only with substantial friction, making their manipulation difficult. Consequently, prior reports of forward‐viewing ERCP in this setting have often been limited to plastic stents and ENBD.

The covered multihole SEMS used in this case (HANAROSTENT Biliary Multihole Benefit) has an ultra‐slim 5.9‐Fr delivery system that can be advanced through a 3.2‐mm working channel. SEMS placement using a slim delivery system has previously been reported mainly for uncovered SEMS [6]. In contrast, the multihole stent used in this case is a covered SEMS, which may offer clinical advantages such as suppression of tumor ingrowth and potential removability [9, 10]. Therefore, the availability of an ultra‐slim delivery system is clinically meaningful because it enables covered SEMS placement using standard, widely available endoscopes, without the need for large‐channel instruments. In patients with MBO complicated by type I duodenal stenosis, this may expand the endoscopic options for transpapillary drainage.

In conclusion, transpapillary placement of a covered SEMS using a standard FVE in combination with a 5.9‐Fr delivery system may be a useful therapeutic option in selected patients with MBO and type I duodenal stenosis in whom a forward‐viewing endoscope can traverse the stenosis and papillary access is achievable.

## Author Contributions

**Kengo Matsumoto**: conceptualization. **Kengo Matsumoto**, **Daisuke Kondo**, **Satoru Okabe**, and **Akira Doi**: investigation. **Kengo Matsumoto**, **Daisuke Kondo**, and **Satoru Okabe**: resources. **Kengo Matsumoto**: writing – original draft. **Daisuke Kondo**, **Satoru Okabe**, **Naoto Osugi**, **Akira Doi**, **Masashi Yamamoto**, **Shiro Hayashi**, **Koji Fukui**, and **Tsutomu Nishida**: writing – review & editing. **Shiro Hayashi**, **Koji Fukui**, and **Tsutomu Nishida**: supervision.

## Funding

The authors have nothing to report.

## Ethics Statement

Ethics approval was not required for this case report in accordance with institutional policies.

## Consent

Patient consented.

## Conflicts of Interest

The authors declare no conflicts of interest.
